# City-level process-related CO_2_ emissions in China 2000–2021

**DOI:** 10.1038/s41597-025-05782-3

**Published:** 2025-08-15

**Authors:** Sijia Cai, Jinghang Xu, Yuru Guan, Miaomaio Liu, Chang Tan, Jun Bi, Yuli Shan

**Affiliations:** 1 State Key Laboratory of Pollution Control and Resource Reuse, School of the Environment, Nanjing University, Nanjing, 210023 China; 2 School of Geography, Earth and Environmental Sciences, University of Birmingham, Birmingham, B15 2TT UK; 3 Basic Science Center for Energy and Climate Change, Beijing, 100081 China; 4 Department of Earth System Science, Tsinghua University, Beijing, 100084 China; 5 Birmingham Institute of Sustainability and Climate Action (BISCA), University of Birmingham, Birmingham, B15 2TT UK

**Keywords:** Climate-change mitigation, Climate-change mitigation, Energy modelling

## Abstract

As the world’s largest CO_2_ emitter, China needs accurate city-level CO_2_ emission accounts to formulate effective low-carbon policies. However, previous studies mainly accounted for emissions from fossil fuel combustion and overlooked process-related CO_2_ emissions from industrial production (e.g., mineral, chemical, metal products), which account for approximately 13% of China’s total emissions. In this study, we built the first time-series dataset of process-related CO_2_ emissions for 289 Chinese cities from 2000 to 2021. The dataset covers 11 industrial products and adheres to the methodology recommended by the Intergovernmental Panel on Climate Change (IPCC). We applied China-specific emission factors and compiled industrial output data from city statistical yearbooks and bulletins. Missing output data were imputed using missForest models. The estimated uncertainty of the process-related emissions in our dataset ranges from 3.87% to 3.91%. Our dataset provides a robust foundation for analyzing emission patterns at the city level and for designing targeted low-carbon policies.

## Background & Summary

Climate change is one of the most urgent challenges, and countries are actively seeking solutions to reduce carbon dioxide (CO_2_) emissions^1,2^. To design effective solutions, accurate and detailed CO_2_ emission inventories are essential^3,4^. According to the Intergovernmental Panel on Climate Change (IPCC), CO_2_ emissions in a city may originate from two main sources: energy-related emissions from fossil fuel combustion and process-related emissions from chemical or physical transformations of raw materials without combustion, such as carbonate decomposition or metal reduction^5^. While energy-related CO_2_ emissions have been extensively studied and widely incorporated into policy-making, largely due to their large share and the availability of mature accounting methods, process-related emissions have received far less attention^6–8^. According to the IPCC, more than 30 industrial processes worldwide generate process-related CO_2_ emissions^5^. Overlooking process-related emissions in policy-making may leave major emission sources (e.g., cement and crude steel) unregulated.

As the world’s largest emitter of CO_2_, China plays a pivotal role in global climate mitigation^9^. In response, China has proposed “dual-carbon” goals—to peak CO_2_ emissions before 2030 and achieve carbon neutrality before 2060. China’s accounting for over 50% of global output in key industrial products—such as cement, flat glass, crude steel, and aluminum^10^— highlighting its role as the global manufacturing hub. The country’s large-scale industrial activities and substantial demand for raw material processing, has led to high levels of process-related emissions^11,12^. According to China’s First Biennial Transparency Report on Climate Change, China’s process-related CO_2_ emissions reached 1,524 million tonnes (Mt) in 2021, making up 13% of the nation’s total emissions^13^. This figure is approximately 3.2 times as large as the total fossil CO_2_ emissions of Russia, the world’s fourth-largest emitter^14^. Given the substantial variation in resource endowment and development stage among Chinese cities, the contribution of process-related CO_2_ emissions also varies across cities^15,16^. If these emissions are not properly accounted for, city-level carbon inventories may underestimate total emissions.

In recent years, scholars have increasingly recognized the importance of accurately accounting for process-related CO_2_ emissions. However, existing studies face three major limitations. First, most previous studies usually use global default emission factors recommended by the IPCC, such as those used by Cui *et al*.^17^ and Hu *et al*.^18^. However, these default emission factors can not reflect country-specific industrial processes, technologies, and raw material structures. China-specific emission factors are substantially lower than IPCC default values—by 79% for ferroalloys and 65% for plate glass^5,19,20^. As a result, applying global default factors to China may lead to overestimation of emissions from certain products. Second, most existing studies focus on the national scale, making it difficult to capture city-level differences in emission trends and patterns. Studies such as those by Birgit *et al*.^21^ and Tan *et al*.^22^ provide only national-level estimates for regions like the EU or China. Third, the coverage of industrial product types remains limited. Even at the national level, many studies focus only on a small number of representative products. For example, Xiao *et al*. estimated process-related emissions for a single industrial product in Russia^23^, while Liu included five products in China^24^. Systematic accounting across a broader range of industrial sectors is still lacking, making it difficult to fully reflect the sectoral structure of process-related emissions. Fourth, although some recent studies have attempted city-level estimations, they have primarily focused on the cement production while neglecting other industrial products. Examples include the work by Guo *et al*.^25^ and Shan *et al*.^6^. Such narrow scope limits our understanding of the full landscape of process-related emissions at the city level.

A key challenge in constructing a city-level inventory of process-related CO_2_ emissions lies in the limited availability of complete industrial output data and country-specific emission factors. To address these issues, this study undertook comprehensive data compilation and preprocessing, an extensive literature review, and an uncertainty analysis. Specifically, we compiled output data for 11 products across 289 Chinese cities for the period 2000–2021. Missing values were imputed using four missForest models to ensure data completeness and consistency. The selected products—cement, plate glass, calcium carbide, ethylene, ammonia, soda ash, crude steel, ferroalloy, aluminum, lead, and zinc—account for over 77% of China’s process-related CO_2_ emissions, and the 289 cities represent more than 90% of the national gross domestic product (GDP)^13^. Several other industrial products (e.g., lime, pig iron, carbon black, fluorochemicals, and ceramics) were excluded due to extensive data gaps at the city level. In parallel, we conducted an extensive review of the literature to collect country-specific emission factors that better reflect China’s industrial processes. To evaluate the robustness of the emission estimates, we incorporated the coefficients of variation (CVs) for both activity data and emission factors into a Monte Carlo simulation to quantify the uncertainty in the CO_2_ emission estimates.

Building on this effort, we constructed the first long-term, consistent dataset covering 289 Chinese cities. To ensure transparency, our dataset includes not only the estimated emissions but also the underlying industrial output data used in the calculations, comprising a total of 139,876 records. Our dataset, therefore, provides robust data support for city-level low-carbon policy design and implementation.

## Methods

### Scope and accounting boundary

This study estimates process-related CO_2_ emissions from 11 industrial products across 289 Chinese cities over the period 2000–2021. These products span three major industrial sectors: the mineral industry (cement and plate glass), the chemical industry (calcium carbide, ethylene, ammonia, and soda ash), and the metal industry (crude steel, ferroalloy, aluminum, lead, and zinc).

**Fig. 1:**
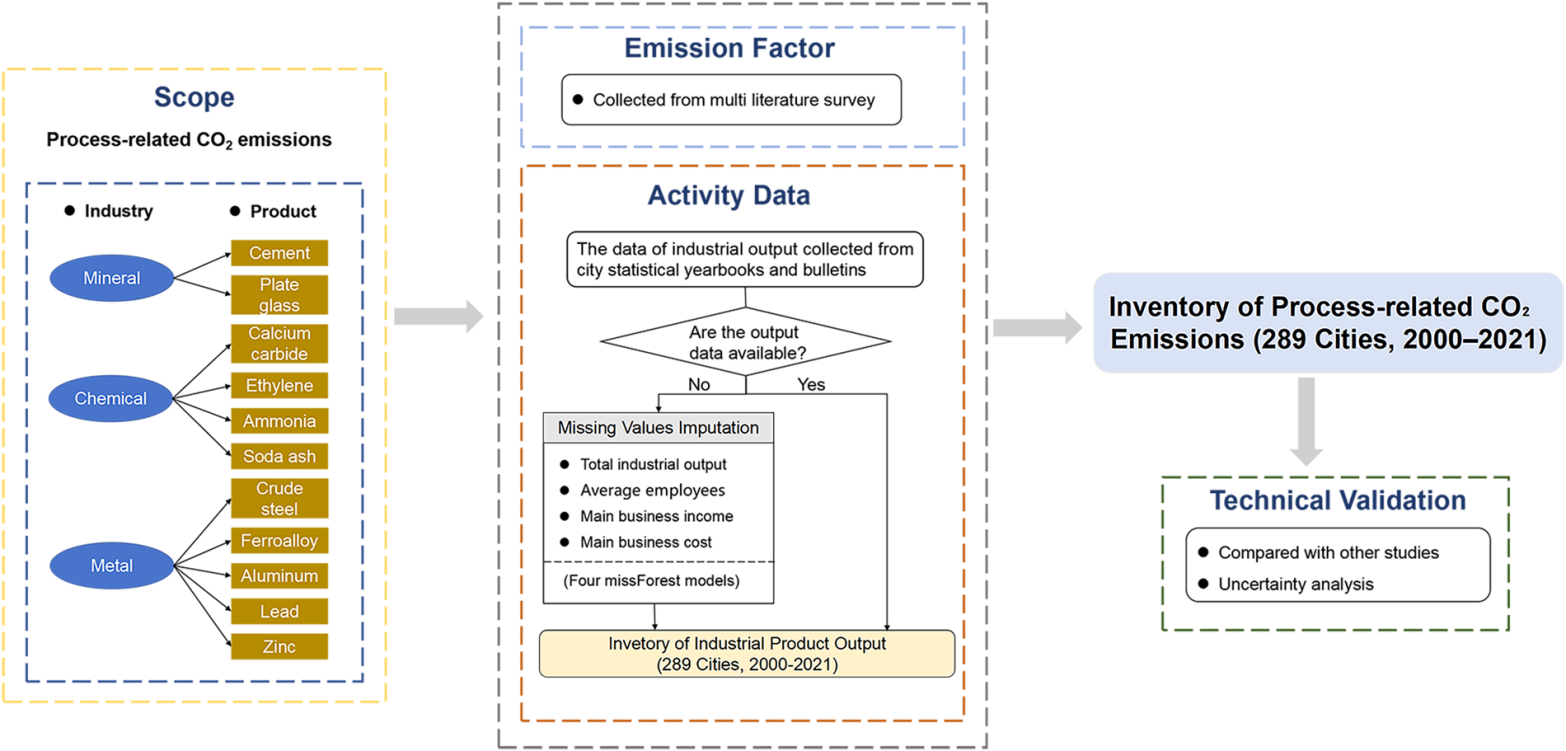
Methodological framework for annual city-level territorial CO_2_ emissions from industrial processes.

To perform the estimation, we adopt the IPCC-recommended administrative territorial-based approach, which accounts for emissions generated within the geographical boundaries of each city. Figure 1 presents the framework used to construct the annual city-level inventory of process-related CO_2_ emissions.

### Process-related CO_2_ emission accounting approach

The emission factor approach is currently the most widely used method for estimating CO_2_ emissions. It calculates emissions by multiplying the activity data by the corresponding emission factors, making it suitable for large-scale assessments and for contexts where direct monitoring data are unavailable^22,25^. The IPCC provide two approaches for selecting emission factors: Tier 1, which uses global default values, and Tier 2, which applies country-specific emission factors that better reflect local production processes^5,26^. To enhance the accuracy and reliability of our estimates, this study adopts the Tier 2 approach for all 11 industrial products, incorporating China-specific emission factors. The general formula used for emission estimation is presented as follows:

1 $${E}_{i}={{AD}}_{i}\times {{EF}}_{i}$$

where ${E}_{i}$ represents CO_2_ emissions from product *i*. ${{AD}}_{i}$ represents the activity data (i.e., the output of product *i*), and ${{EF}}_{i}$ is the emission factor, indicating the amount of CO_2_ emitted per tonne of product *i*.

For certain products such as soda ash, crude steel, lead, and zinc, we follow the methodology proposed by Yu *et al*.^27^, using a weighted average of emission factors based on the share of different production processes to improve estimation accuracy. The specific calculation methods for these products are described below:

1. *Soda ash:* Among the three soda ash production processes, only the natural soda process generates CO_2_ emissions. According to the China Soda Ash Industry Association (CSIA), this process accounts for 5% of China’s total soda ash production. Emissions are calculated as:
  2 $${E}_{{soda\; ash}}={{AD}}_{{soda\; ash}}\ast 5 \% \ast {{EF}}_{{soda\; ash}}$$
  where ${{AD}}_{{soda\; ash}}$ is the total output of soda ash, and ${{EF}}_{{soda\; ash}}$ is the emission factor for soda ash.
2. *Crude steel:* Process-related CO_2_ emissions from crude steel production arise from carbon oxidation during the conversion of pig iron to crude steel, and limestone decomposition used as a flux in steelmaking. Crude steel is primarily produced via two technological routes: the blast furnace-basic oxygen furnace (BF-BOF)and the electric arc furnace (EAF). The first component is calculated by assessing the difference in carbon content between pig iron (4.1%) and crude steel (0.25%), and converting to CO_2_ using the molecular weight ratio (44/12):
  3 $${E}_{{crude\; steel}(1)}=({{AD}}_{{crude\; steel}({BF}-{BOF})}+{{AD}}_{{crude\; steel}({EAF})})\ast (4.1 \% -0.25 \% )\ast \frac{44}{12}$$
  where ${{AD}}_{{crude\; steel}({BF}-{BOF})}$ and ${{AD}}_{{crude\; steel}({EAF})}$ are the outputs of crude steel from each respective process.
  The second component arises from limestone decomposition. The specific limestone consumption is 0.3 tonnes per tonne of crude steel for BF–BOF and 0.064 tonnes for EAF^28^. Emissions from this source are calculated as:
  4 $${E}_{{crude\; steel}(2)}=({{AD}}_{{crude\; steel}({BF}-{BOF})}\ast 0.3+{{AD}}_{{crude\; steel}\left({EAF}\right)}\ast 0.064)\ast {{EF}}_{{limestone}}$$
  where ${{EF}}_{{limestone}}$ is the emission factor for limestone decomposition.
3. *Lead:* Lead production in China consists of primary and secondary sources. Among primary sources, the imperial smelting furnace (ISF) accounts for 2.5% of output, while direct smelting (DS) contributes 97.5%^29^. Secondary lead is primarily recovered via battery processing (BP) from used lead-acid batteries. The total emissions from lead production are calculated as:
  5 $${E}_{{lead}}={{AD}}_{{lead}}\ast 2.5 \% \ast {{EF}}_{{lead}({ISF})}+{{AD}}_{{lead}}\ast 97.5 \% \ast {{EF}}_{{lead}({DS})}+{{AD}}_{{lead}({secondary})}\ast {{EF}}_{{lead}({BP})}$$
  where ${{AD}}_{{lead}}$ and ${{AD}}_{{lead}({secondary})}$ denote total and secondary lead outputs, respectively. ${{EF}}_{{lead}\left({ISF}\right)}$, ${{EF}}_{{lead}\left({DS}\right)}$ and ${{EF}}_{{lead}\left({IBP}\right)}$ are the emission factors for ISF, DS, and BP processes, respectively.
4. *Zinc:* In China, zinc is mainly produced through ISF, electro-thermic distillation (ETD), and pyrometallurgical processing (PMP). Only the ISF process generates CO_2_ emissions, accounting for 5% of national zinc output^29^. Thus, emissions are calculated as:

6 $${{\rm{E}}}_{{\rm{zinc}}}={{\rm{AD}}}_{{zinc}}\ast 5 \% \ast {{\rm{EF}}}_{{zinc}({ISF})}$$

where ${{AD}}_{{zinc}}$ is the total zinc output, and ${{EF}}_{{zinc}\left({ISF}\right)}$ is the emission factor for zinc production using the ISF process.

### Activity data and emission factors

In this study, activity data refer to the annual output of 11 industrial products from industrial enterprises above designated size across 289 cities. These data were collected from city-level statistical yearbooks and bulletins^30^. Each city’s yearbook was accessed via its official government website (e.g., http://tjj.beijing.gov.cn/; http://tjj.nanjing.gov.cn/). However, due to inconsistencies in data quality at the city level, approximately 2% of the output data are missing. To overcome this problem, we applied missForest, a nonparametric imputation method based on random forests, for data interpolation^31^. This method is well-suited for imputing both continuous and categorical variables, particularly in datasets with complex interactions and nonlinear relationships^32,33^.

To improve imputation accuracy, we developed four separate missForest models based on the industry classification introduced earlier. Specifically, the mineral and chemical sectors each used a single model, while the metal sector was further divided into ferrous and non-ferrous smelting and rolling. This sectoral division is informed by well-established distinctions in production technologies, statistical reporting practices, and data distributions between ferrous and non-ferrous metals, which may lead to divergent patterns of missingness and variation in output data. Each model incorporated sector-specific predictors, including total industrial output value, average number of employees, main business income, main business cost, and city type. The first four variables were obtained from city-level statistical yearbooks^30^, and city types were derived using K-means clustering based on the employment and GDP structure.

For emission factors, we conducted a comprehensive review of the existing literature and compiled values from multiple sources (see Table 1). Due to the lack of empirical data at the city level and over time, these emission factors are assumed to be uniform and time-invariant throughout the study period and across all cities.

**Table 1:** Emission factors for 11 industrial products.

Industrial product	Production process	Value	Unit	Source
Cement	/	0.29	tCO_2_/t	Liu *et al*.^19^
Plate glass	/	0.0737	tCO_2_/t	Hu *et al*.^20^
Calcium carbide	/	1.15	tCO_2_/t	NDRC, 2011^46^
Ethylene	/	2.25	tCO_2_/t	IPCC, 2006^5^
Ammonia	/	2.97	tCO_2_/t	Yu *et al*.^27^
Soda ash	Natural soda	0.14	tCO2/t	IPCC, 2006^5^ IPCC, 2006^5^ IPCC, 2006^5^
	Solvay soda	0.00	tCO_2_/t	
	Hou’s soda	0.00	tCO2/t	
Limestone use	/	0.43	tCO_2_/t Limestone	NDRC, 2011^46^
Carbon content of pig iron	/	4.10	%	NDRC, 2011^46^
Carbon content of steel	/	0.25	%	NDRC, 2011^46^
Ferroalloy	/	0.28	tCO_2_/t	NDRC, 2020^47^
Aluminum	/	1.50	tCO2/t	NDRC, 2020^47^
Lead	Imperial smelting furnace	0.66	tCO_2_/t	Sjardin, 2003^48^
	Direct smelting	0.25	tCO2/t	Sjardin, 2003^48^
	Battery processing	0.20	tCO_2_/t	Sjardin, 2003^48^
Zinc	Imperial smelting furnace	3.12	tCO2/t	Sjardin, 2003^48^
	Electro-thermic distillation	0.00	tCO_2_/t	Sjardin, 2003^48^
	Pyrometallurgical process	0.00	tCO_2_/t	Sjardin, 2003^48^

## Data Record

The dataset is available as open access via Figshare (10.6084/m9.figshare.29085002.v3)^34^ and contains 139,876 records on process-related CO_2_ emissions and industrial product output. Specifically:

- 69,938 records document the output of 11 industrial products in 289 cities from 2000 to 2021 (File “China industrial product output inventory at the city level”);
- 69,938 records document process-related CO_2_ emissions for the same 11 products across 289 cities from 2000 to 2021 (File “China process-related CO_2_ emission inventory at the city level”).

The CO_2_ emission inventory follows a standardized format and is organized across multiple spreadsheet sheets, with each sheet corresponding to a specific year from 2000 to 2021. Within each sheet, the data are structured into 12 columns and 289 rows. The columns represent process-related emissions for each of the 11 industrial products, along with the total process-related emissions for each city. The rows represent the 289 cities. Each entry records the emissions associated with producing a specific product in a given city.

## Technical Validation

### Statistical overview of the dataset

Between 2000 and 2021, process-related CO_2_ emissions from the 11 industrial products showed a notable increase. Total emissions rose from 294 Mt in 2000 to 1,110 Mt in 2021, with an average annual growth rate of 6.5% and an annual increase of 39 Mt. Among the three industries, the mineral industry contributed the largest absolute increase in emissions, the metal industry exhibited the highest relative growth rate, while the chemical industry experienced comparatively slower growth.

**Fig. 2:**
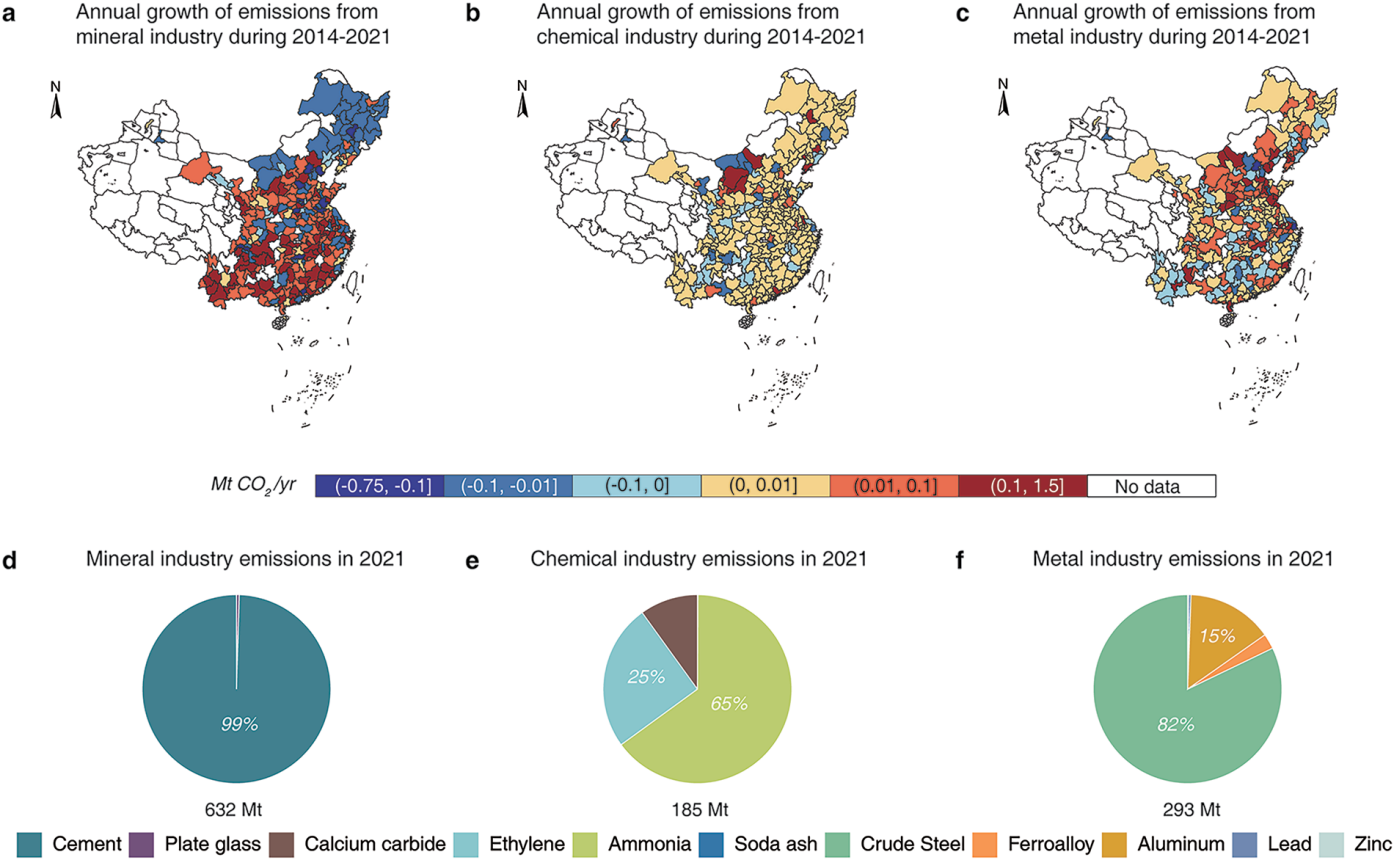
Spatial distribution and structure of process-related CO_2_ emissions. (**a**–**c**) Average annual growth of process-related CO_2_ emissions in the mineral, chemical, and metal industries from 2014 to 2021. Warmer colors (yellow to red) indicate emission increases, while cooler colors (light blue to blue) represent emission reductions. d-f Process-related CO_2_ emissions composition of the mineral, chemical, and metal industries in 2021.

The mineral industry was the largest contributor to process-related CO_2_ emissions. Emissions increased from 168 Mt in 2000 to 632 Mt in 2021, with an average annual increase of 22 Mt and a growth rate of 6.5%. Figure 2a shows that between 2014 and 2021, emission hotspots in this industry gradually shifted from northern and northeastern China to southern and southwestern regions. This spatial shift is largely driven by rapid economic growth and rising construction demand in southern provinces after 2014^35,36^. The fastest-growing cities were Meizhou in Guangdong province (0.4 Mt/yr), Sanming in Fujian (0.36 Mt/yr), and Chengde in Sichuan (0.32 Mt/yr). In contrast, Shijiazhuang in Hebei (−0.7 Mt/yr), Xuzhou in Jiangsu (−0.59 Mt/yr), and Changchun in Jilin (−0.39 Mt/yr) experienced substantial declines. Figure 2d presents the emission composition of the mineral industry in 2021, showing that cement accounted for 99%, while the contribution from plate glass was relatively minor.

The chemical industry showed slower growth, with total emissions increasing from 93 Mt in 2000 to 185 Mt in 2021. The average annual growth rate was 3.3%, with an annual increase of 4 Mt. As illustrated in Fig. 2b, emission growth was concentrated in northern cities, including Ordos in Inner Mongolia (0.32 Mt/yr), Dalian in Liaoning (0.31 Mt/yr), and Yulin in Shaanxi (0.3 Mt/yr). Meanwhile, emissions declined in Shijiazhuang in Hebei (−0.26 Mt/yr), Liuzhou in Guangxi (−0.13 Mt/yr), and Hengshui in Hebei (−0.12 Mt/yr). Figure 2e shows the 2021 emission composition: ammonia contributed 65%, ethylene 25%, while calcium carbide and soda ash accounted for smaller shares.

The metal industry experienced the largest relative growth, with emissions rising from 32 Mt in 2000 to 293 Mt in 2021. The average annual growth rate reached 11.1%, with an annual increase of 12 Mt. As shown in Fig. 2c, between 2014 and 2021, emission growth was concentrated in resource-intensive cities. The fastest-growing cities were Tangshan in Hebei (1.39 Mt/yr), Binzhou in Shandong (0.86 Mt/yr), and Baotou in Inner Mongolia (0.48 Mt/yr). In contrast, emissions declined in Shanghai (−0.16 Mt/yr), Beijing (−0.1 Mt/yr), and Tianjin (−0.09 Mt/yr). Figure 2f presents the 2021 emission composition for the metal industry: crude steel accounted for 82%, followed by aluminum (15%), while lead, zinc, and other metals contributed relatively minor shares.

### Uncertainty analysis

Uncertainty analysis is a key tool for evaluating the quality of CO_2_ emission inventories^37,38^. According to the IPCC, uncertainties primarily originate from activity data and emission factors^5^. Two commonly used methods for uncertainty assessment in previous studies are error propagation and Monte Carlo simulation. Although error propagation is relatively easy to implement, it relies on the assumptions of linearity and normally distributed input variables. These assumptions make it unsuitable when input parameters have large uncertainties or skewed distributions^27^. In contrast, Monte Carlo simulation does not depend on such assumptions and can flexibly accommodate probability density functions of any shape and range, providing a more accurate representation of input uncertainties and their propagation in emission estimates^39^. Therefore, this study adopts the Monte Carlo method to assess uncertainties in the estimation of process-related CO_2_ emissions.

In our analysis, we assigned a coefficient of variation (CV) of 5% to activity data obtained from official statistical yearbooks and bulltins^27,40^. For data imputed using missForest models, the CV was set at 15%^5,41^. Emission factor CVs follow the values recommended by the IPCC (see Table 2)^5^. Emissions from ferroalloy production were excluded from the uncertainty analysis due to the complexity of its carbon sources (e.g., petroleum coke, graphite, biomass), which makes the quantification of its emission factor uncertainty particularly challenging.

**Table 2:** CVs for emission factors.

Industrial product	CV (%)
Cement	5.5
Glass	30
Calcium carbide	10
Ethylene	30
Ammonia	6
Soda ash	0
Limestone use	6
Crude Steel	5
Aluminum	10
Lead	50
Zinc	50

Probability distributions were derived based on a comprehensive literature review^42,43^. When the uncertainty range is less than ±60%, it is typically assumed to follow a normal distribution^44,45^. Accordingly, we assumed normal distributions for both activity data and emission factors. A total of 20,000 random samples were generated to estimate the uncertainty range of process-related CO_2_ emissions at the 95% confidence level. The results show that the total inventory uncertainty ranges from −3.87% to +3.91%. At the city level, Zhoushan (Zhejiang province) exhibited the highest uncertainty (−18.74% to +18.78%), while Ürümqi (Xinjiang Uygur Autonomous Region) had the lowest (−1.90% to +1.93%).

To evaluate the potential impact of excluding ferroalloy emissions, we conducted a sensitivity analysis assuming a conservative CV of 50%, which showed that their inclusion would alter the total uncertainty range by less than 0.1 percentage point.

### Comparison with existing emission estimates

**Fig. 3:**
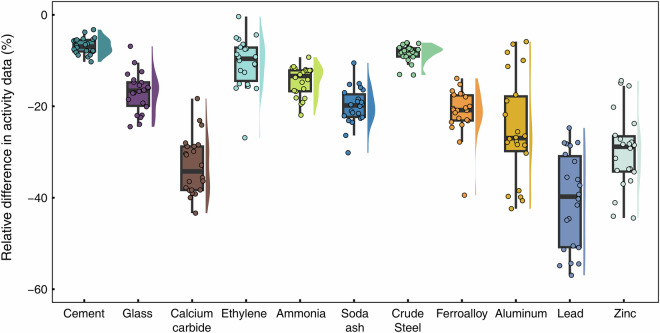
Relative differences in activity data between this study and national statistics. The relative difference in activity data is calculated as (this study’s data/national data) − 1. A negative value indicates that the activity data collected in this study are lower than the national statistics.

In addition to uncertainty analysis, we further evaluated the reliability of our activity data and emission estimates by comparing them with national statistics and results from previous studies. First, we compared the collected output data for 11 industrial products with national-level statistics (see Fig. 3). Since this study covers 289 cities and not the entire country, the activity data for all 11 products are generally lower than national statistics. This discrepancy is primarily due to the exclusion of cities not included in our study.

Among all products, the outputs of calcium carbide, aluminum, lead, and zinc in our dataset are more than 20% lower than the corresponding national totals. These products are likely produced in smaller or less-developed cities that are not part of our study. Additionally, our data are primarily sourced from statistical yearbooks and bulletins, which only include industrial enterprises above the designated size. As a result, outputs from small enterprises are often excluded, leading to further underreporting. In contrast, the reported outputs of cement, plate glass, ethylene, ammonia, soda ash, crude steel, and ferroalloy in this study are only slightly lower than the national totals. This consistent underestimation suggests that the dataset is reliable—it does not overreport industrial output, which is crucial for ensuring the accuracy of CO_2_ emission estimates.

**Fig. 4:**
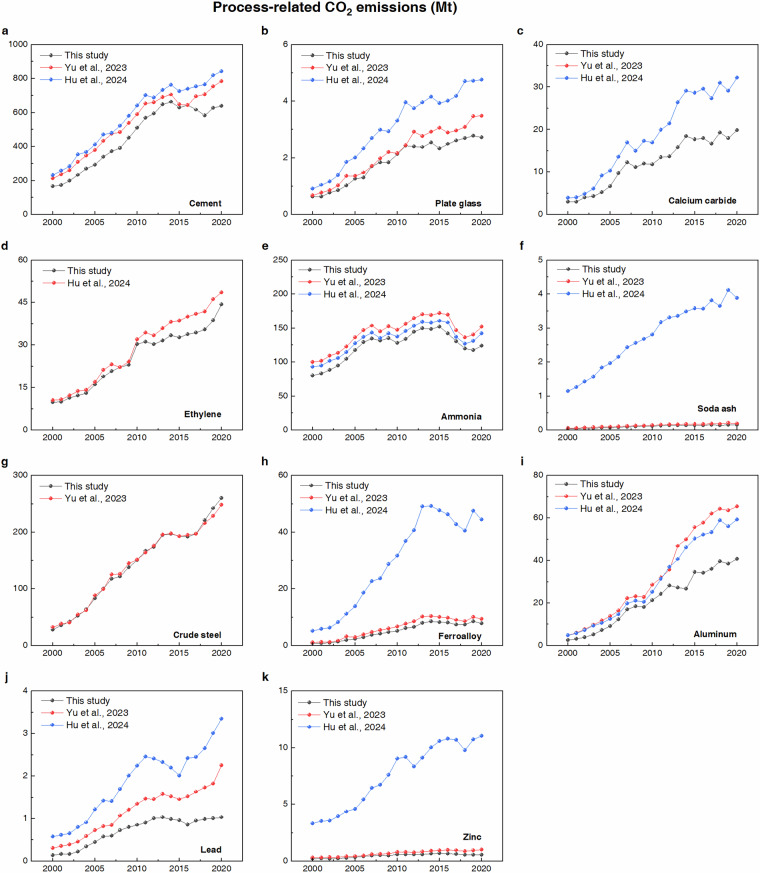
Process-related CO_2_ emissions from 11 industrial products compared with other studies. Please note that the ranges of the y-axis are different in each subplot.

To further assess the accuracy of our emission estimates, we compared them with those reported by Yu *et al*.^27^ and Hu *et al*.^18^, two of the most comprehensive national-scale studies on China’s process-related CO_2_ emissions. Since no existing dataset provides city-level coverage of multiple industrial products, these national-level estimates serve as the most appropriate reference point for validating our results. As shown in Fig. 4, our relatively lower emission estimates reflect a narrower spatial coverage by design, rather than an underestimation. Unlike national-level studies, our study improves spatial resolution by providing city-level CO_2_ emission estimates. Furthermore, the use of localized emission factors and more complete output data further improves the accuracy and completeness of the estimates.

In the mineral industry (Fig. 4a–b), our estimates for cement and plate glass are consistently lower than those of Yu *et al*. and Hu *et al*. during 2000–2020. For cement, this is mainly due to methodological variation—our study calculates emissions based on cement output, whereas both Yu *et al*. and Hu *et al*. use clinker-based estimates. On average, our cement emissions are 16% lower than those of Yu *et al*. and 22% lower than those of Hu *et al*. For plate glass, both our study and Yu *et al*. both apply a China-specific emission factor (0.07 t CO_2_/t), while Hu *et al*. adopts the default value (0.1 t CO_2_/t), resulting in estimates that are 39% higher than ours. The 13% lower estimate compared to Yu *et al*. is mainly attributable to cities not included in our sample.

In the chemical industry (Fig. 4c–f), our emission estimates for calcium carbide, ethylene, ammonia, and soda ash are consistently lower than those of Yu *et al*. and Hu *et al*. All three studies use the same emission factor for calcium carbide (1.15 t CO_2_/t), so the 33% lower estimate in our study is due to unstudied cities. Similarly, we use the same emission factor for ethylene (2.25 t CO_2_/t) as Hu *et al*., yet our results are approximately 10% lower. For ammonia, both our study and Yu *et al*. use a China-specific emission factor (2.97 t CO_2_/t), while Hu *et al*. adopts the lower default emission factor (2.77 t CO_2_/t). As a result, our estimates are 14% lower than Yu *et al*. and 8% lower than Hu *et al*. In the case of soda ash, although all three studies adopt the same emission factor (0.67 t CO_2_/t), Hu *et al*. does not account for the fact that only 5% of China’s soda ash is produced via the natural soda process, which is the only one that generates CO_2_ emissions. This omission leads to substantial overestimation—Hu *et al*.’s estimate is 96% higher than ours, while ours are 20% lower than Yu *et al*.’s.

In the metal industry (Fig. 4g–k), our emission estimates for ferroalloy, aluminum, lead, and zinc are consistently lower than those of Yu *et al*. and Hu *et al*., whereas our estimates for crude steel are slightly higher in certain years. The higher estimates for crude steel are due to data limitations: we lack city-level data on pig iron consumption and the technological breakdown of crude steel production via BF–BOF and EAF routes. Therefore, we use total crude steel output as a proxy and apply national-level process shares to estimate emissions. For ferroalloy, aluminum, and zinc (Fig. 4h,i,k), we use the same emission factors as Yu *et al*. (0.28, 1.5, and 3.12 t CO_2_/t respectively). However, our results are 21%, 35%, and 29% lower, respectively, due to differences in spatial coverage. By contrast, Hu *et al*. adopts default emission factors (1.3, 1.6, and 1.72 t CO_2_/t), which do not align well with China’s actual industrial practices. Consequently, our estimates are 83%, 29%, and 94% lower than those of Hu *et al*., respectively. For lead (Fig. 4j), we follow the same methodology as Yu *et al*., applying a weighted average based on different smelting processes. But due to the lack of city-level process shares, we rely on national-level proportions. Hu *et al*., on the other hand, uses a single default emission factor without distinguishing between smelting processes, resulting in 63% higher emissions compared to our study.

## Data Availability

All code used to construct the industrial product output dataset is published under https://github.com/sijcai/Industrial-Product-Output. The code is written in R 4.3.0.
